# Association between peripheral endothelial function and myocardial perfusion in patients with coronary artery disease

**DOI:** 10.1093/ehjimp/qyae010

**Published:** 2024-02-13

**Authors:** Niklas Vartiainen, Juha E K Hartikainen, Tiina M Laitinen, Paavo-Ilari Kuikka, Hanna Mussalo, Tomi P Laitinen

**Affiliations:** Department of Clinical Physiology and Nuclear Medicine, Kuopio University Hospital, Puijonlaaksontie 2, 70210 Kuopio, Finland; Heart Center, Kuopio University Hospital, Kuopio, Finland; Department of Clinical Physiology and Nuclear Medicine, Kuopio University Hospital, Puijonlaaksontie 2, 70210 Kuopio, Finland; Department of Clinical Physiology and Nuclear Medicine, Kuopio University Hospital, Puijonlaaksontie 2, 70210 Kuopio, Finland; Department of Clinical Physiology and Nuclear Medicine, Kuopio University Hospital, Puijonlaaksontie 2, 70210 Kuopio, Finland; Department of Clinical Physiology and Nuclear Medicine, Kuopio University Hospital, Puijonlaaksontie 2, 70210 Kuopio, Finland; Institute of Clinical Medicine, University of Eastern Finland, Kuopio, Finland

**Keywords:** endothelial function, flow-mediated dilation, vasodilation, myocardial blood flow, adenosine

## Abstract

**Aims:**

Endothelial dysfunction is a systemic disorder and risk factor for atherosclerosis. Our aim was to assess whether there is a relation between peripheral endothelial function and myocardial perfusion in patients with coronary artery disease (CAD).

**Methods and results:**

We prospectively studied 54 patients, who had a positive result for obstructive CAD in coronary CT angiography. Myocardial perfusion (^15^O)H_2_O positron emission tomography was imaged at rest and during adenosine-induced maximal vasodilation. Peripheral endothelial function was assessed by measuring flow-mediated dilation (FMD) with ultrasound from the left brachial artery. There was a statistically significant correlation between FMD and global hyperaemic myocardial blood flow (MBF; *r* = 0.308, *P* = 0.023). The correlation remained statistically significant when controlling for gender, height, and diastolic blood pressure at rest (*r* = 0.367, *P* = 0.008). Receiver operating character analysis, however, yielded an area under curve of only 0.559 (*P* = 0.492) when FMD was used to predict reduced MBF (below 2.3 mL/g/min). Patients with significantly decreased MBF (*n* = 14) underwent invasive coronary angiography. FMD showed an inverse correlation with the severity of the most significant stenosis (*r* = −0.687, *P* = 0.007).

**Conclusion:**

Peripheral endothelial function is related with hyperaemic MBF and with the severity of CAD in invasive coronary angiography. Due to insufficient sensitivity and specificity in the identification of reduced MBF, FMD is not suitable for clinical practice at the individual level. However, it works at the population level as a research tool when assessing endothelial dysfunction in patients with CAD.

## Graphical Abstract

**Graphical Abstract:**
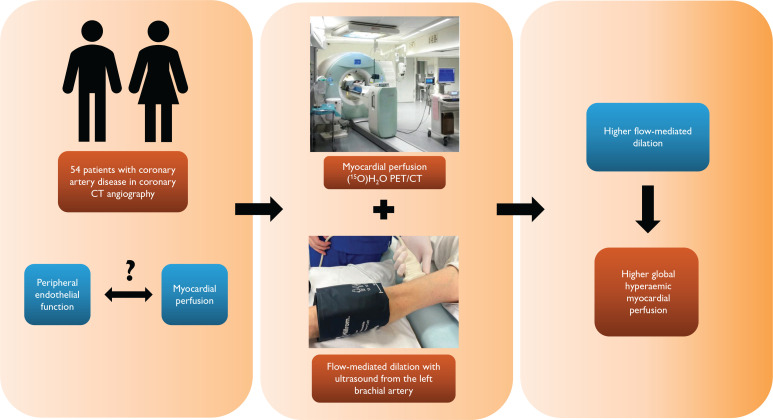

## Introduction

Endothelial dysfunction plays an important role in atherosclerosis.^1^ It is crucial in the development of coronary artery disease (CAD) and is associated with traditional cardiovascular risk factors, such as hypertension, hypercholesterolaemia, and smoking.^2–4^ Additionally, it is a risk factor for adverse cardiovascular events.^5–7^

Endothelial function can be assessed non-invasively by measuring flow-mediated dilation (FMD) from a peripheral artery.^8^ FMD has been identified as an independent predictor of cardiovascular events in patients without cardiovascular diseases.^9^ The association between FMD and severity of CAD has also been studied to some extent. There are some reports on FMD and coronary calcium score,^10–12^ but only one study has assessed the association between FMD and severity of CAD based on coronary angiography.^13^ A few studies have shown that impaired FMD is related with myocardial ischaemia assessed with semiquantitative single-photon emission computed tomography.^14–16^

Myocardial perfusion positron emission tomography (PET) can be used to determine the functional significance of epicardial coronary artery lesions.^17^ However, the utility of quantitative PET imaging goes beyond the assessment of epicardial coronary artery stenoses, as it also reflects the function of coronary microcirculation.^18^ During PET imaging, maximal coronary vasodilation and consecutive hyperaemia are achieved by intravenous infusion of adenosine. Adenosine-induced vasodilation is proposed to be at least partly mediated by endothelial function, but the subject has remained controversial.^19–23^ Since some of these studies have shown that adenosine has an endothelium-dependent effect, it could be argued that adenosine-induced hyperaemic blood flow could be used as a marker of coronary endothelial function.

There are publications on the association between FMD and myocardial flow reserve (MFR) in patients with dilated cardiomyopathy and Fabry’s disease,^24,25^ but to the best of our knowledge, no studies have assessed the relation between FMD and quantitative MBF in patients with CAD. In this study, we aimed to assess the association between peripheral endothelial function and quantitative MBF during adenosine-induced vasodilation in this specific patient population.

## Materials and methods

### Study population

We prospectively studied 54 patients referred for coronary hybrid imaging at Kuopio University Hospital due to suspicion of CAD. Coronary hybrid imaging included coronary CT angiography (CCTA) and, if suggestive of obstructive CAD, *ad hoc* myocardial perfusion PET. Each patient had a luminal stenosis of ≥50% in at least one coronary artery in the CCTA and was subsequently imaged with myocardial perfusion PET. The exclusion criteria included prior history of CAD, chronic heart failure, permanent atrial fibrillation, and atrial flutter. From the patient flow, an average of one patient per week was invited to participate in an additional study visit that included physiological measurements, of which this article examines brachial ultrasound imaging in relation to myocardial perfusion PET findings. For practical reasons, participation in the study was limited, however, avoiding selection for reasons other than the aforementioned exclusion criteria.

The patients’ clinical characteristics including symptoms, comorbidities, and risk factors were obtained during their hospital visit for hybrid imaging. The pre-test probability of CAD was assessed according to the 2019 European Society of Cardiology guidelines on chronic coronary syndromes.^26^

Prior to participation, the patients were informed of the study protocol, and they provided their informed consent. This study adhered to the Declaration of Helsinki and was approved by the local ethics committee.

### CCTA and myocardial perfusion PETT

The patients were instructed to abstain from caffeine, alcohol, and smoking for at least 12, 48, and 4 h prior to coronary hybrid imaging, respectively. They also had to avoid physical exertion for at least 24 h prior to the imaging.

CCTA images were obtained using the Biograph Vision CT scanner (Siemens Healthineers, Erlangen, Germany). Before CT imaging, intravenous metoprolol at a maximum dose of 20 mg was administered to slow down the heart rate below 65 beats/min. Additionally, 1.25 mg of sublingual isosorbide nitrate was given. The imaging began with the captured native CT images to assess the coronary artery calcium scores according to Agatston *et al*.^27^ Next, prospective, ECG-gated and contrast-enhanced images were obtained during mid-diastole. CCTA was interpreted to be positive for obstructive CAD when either non-calcified or calcified plaque causing stenosis of ≥50% was observed in at least one coronary artery.

Myocardial perfusion imaging was performed *ad hoc* on the same day after the CCTA with a PET/CT scanner, either Biograph mCT or Biograph Vision (Siemens Healthineers, Erlangen, Germany). The protocol included dynamic imaging at rest and during adenosine-induced hyperaemia with (^15^O)H_2_O tracer. Adenosine was infused intravenously at a rate of 140 μg/kg/min, and the activity of (^15^O)H_2_O amounted to 500–800 MBq based on the patient’s weight. A low-dose CT was used for attenuation correction. Myocardial perfusion was quantified with Carimas software (Turku PET Centre, Turku, Finland) by using a single tissue compartment model.^28^ Using a 17-segment model, MBF was quantified globally and separately for each myocardial segment in mL/g/min. Hyperaemic perfusion was considered reduced when at least one segment had an MBF of <2.3 mL/g/min.^29^ Global MFR was determined as the ratio between global hyperaemic and global resting MBF.

### FMD

FMD was assessed on a separate day (range 1–29 days, median 6 days) after the CCTA and myocardial perfusion PET. The patients were instructed to continue their current medication prior to the measurement. FMD was measured from the left brachial artery by using EPIQ 7 ultrasound system equipped with an 18 MHz linear transducer (Philips, Amsterdam, Netherlands). Using the B-mode, a longitudinal image of the brachial artery ∼1–5 cm proximal to antecubital fossa was obtained, and measurement of the end-diastolic artery diameter was performed at rest and during reactive hyperaemia. Reactive hyperaemia was induced by inflating a pneumatic cuff set around the left forearm to a pressure of 250 mmHg for 4.5 min, as described by Juonala *et al*.^30^ After cuff deflation, artery diameter measurements were repeated every 20 s for 2 min. FMD was determined as the greatest percentual change between the baseline diameter and the diameter during reactive hyperaemia. Test–retest reproducibility of FMD has been reported earlier in the publication by Juonala *et al*.^30^

### Invasive coronary angiography

Patients (*n* = 14) with significantly reduced MBF in PET were further referred for invasive coronary angiography. Obstructive CAD was defined as diameter stenosis of ≥50%. The severity of CAD was classified based on the number of diseased main coronary arteries as 1-, 2-, or 3-vessel disease and the percentage of the most significant stenosis.

### Statistical analysis

SPSS Statistics version 27 (IBM, Armonk, New York, USA) was used for statistical analysis. Non-parametric statistical tests were used since FMD and most of the other parameters were not normally distributed. Spearman’s rank correlation coefficient was used to determine the correlation between FMD and other continuous parameters. Mann–Whitney *U* test was performed to compare continuous data between groups. Partial rank correlation was used to perform multivariate correlation analysis. Additionally, a receiver operating character (ROC) analysis was performed to assess how well FMD could predict reduced MBF (i.e. below 2.3 mL/g/min) detected in hyperaemic myocardial perfusion PET. Statistical significance was assumed at *P*-value < 0.05.

## Results

The mean age of the patients was 66 ± 7 years, and 32 (59%) were women (*Table 1*). Hyperaemic perfusion was reduced in at least 1 segment in 16 patients (*Table 2*). Global hyperaemic perfusion was significantly higher in women than in men (3.78 vs. 3.06 mL/g/min, *P* = 0.001). Hyperaemic perfusion correlated with systolic blood pressure at rest (*r* = 0.293, *P* = 0.032), diastolic blood pressure at rest (*r* = 0.305, *P* = 0.025), and patient height (−0.295, *P* = 0.030).

**Table 1:** Clinical characteristics of the study population

	All (*n* = 54)
Women	32 (59)
Age (years)	66 ± 7
Height (cm)	168 ± 9
Weight (kg)	80 ± 15
Body mass index (kg/m^2^)	28 ± 4
Pre-test probability of CAD (%)	20 ± 9
Hypertension	44 (82)
Hypercholesterolaemia	51 (94)
Diabetes mellitus	12 (22)
Ever-smoker	26 (48)
Family history of CAD	40 (74)
Statin	40 (74)
Beta-blocker	23 (43)
ACE inhibitor	10 (19)
Angiotensin II blocker	29 (54)
Calcium blocker	13 (24)
Antithrombotic/anticoagulant therapy	28 (52)
Long-acting nitrate	0 (0)

CAD, coronary artery disease; ACE, angiotensin-converting enzyme.

Values are mean ± standard deviation or *n* (%).

**Table 2:** Findings in CCTA, myocardial perfusion PET, and FMD measurement

	All (*n* = 54)
CCTA	
1-vessel disease (*n*)	33 (61)
2-vessel disease (*n*)	9 (17)
3-vessel disease (*n*)	12 (22)
LM or proximal LAD disease (*n*)	30 (56)
Calcium score	355 ± 761
Myocardial perfusion PET	
Global resting MBF (mL/g/min)	1.11 ± 0.31
Global hyperaemic MBF (mL/g/min)	3.49 ± 0.80
Global MFR	3.29 ± 0.94
MBF < 2.3 mL/g/min (*n*)	16 (30)
FMD measurement	
Baseline systolic blood pressure (mmHg)	139 ± 16
Baseline diastolic blood pressure (mmHg)	88 ± 11
Baseline brachial artery diameter (mm)	3.49 ± 0.65
FMD (%)	5.1 ± 3.8
FMD (mm)	0.17 ± 0.12

CCTA, coronary computed tomographic angiography; LM, left main coronary artery; LAD, left anterior descending coronary artery; PET, positron emission tomography; MBF, myocardial blood flow; MFR, myocardial flow reserve; FMD, flow-mediated dilation.

Values are either mean ± standard deviation or *n* (%).

**Figure 1:**
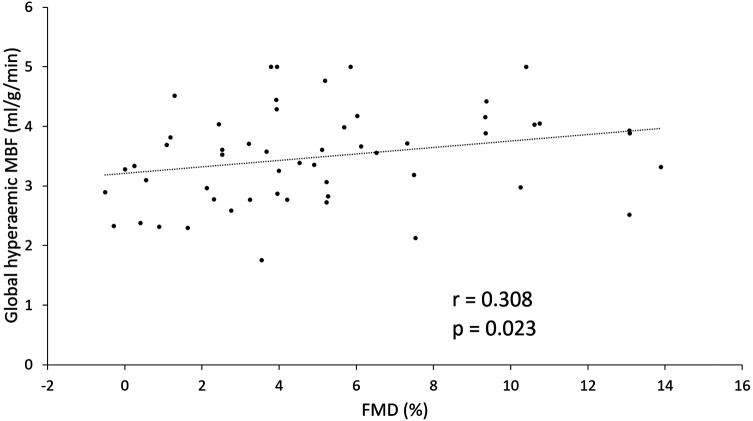
Correlation between FMD (%) and global hyperaemic MBF. FMD, flow-mediated dilation; MBF, myocardial blood flow.

FMD (%) correlated statistically significantly with global hyperaemic MBF (*r* = 0.308, *P* = 0.023) but not with global resting MBF or global MFR (*Table 3*). FMD also correlated positively with diastolic blood pressure at rest (*r* = 0.296, *P* = 0.030). Partial rank correlation analysis showed that the correlation between FMD and global hyperaemic MBF remained statistically significant when controlling for determinants of hyperaemic MBF, i.e. sex, height, and diastolic blood pressure at rest (*r* = 0.367, *P* = 0.008). A scatter plot demonstrating the relation between FMD and global hyperaemic MBF is presented in *Figure 1*.

**Table 3:** Correlation between FMD (%) and other parameters

	Correlation coefficient	*P*-value
Age	−0.053	0.704
Weight	−0.249	0.070
Body mass index	−0.163	0.238
Calcium score	−0.135	0.330
Resting systolic blood pressure	0.159	0.251
Resting diastolic blood pressure	0.296	0.030
Global resting MBF	0.156	0.260
Global hyperaemic MBF	0.308	0.023
Global MFR	0.080	0.564

FMD, flow-mediated dilation; MBF, myocardial blood flow; MFR, myocardial flow reserve.

There was no statistical difference in FMD between patients with normal hyperaemic perfusion and those with reduced MBF (5.3% vs. 4.5%, *P* = 0.459). The ROC analysis that assessed the accuracy of FMD in predicting reduced hyperaemic MBF in PET yielded an area under the curve of 0.559 (*P* = 0.492).

**Figure 2:**
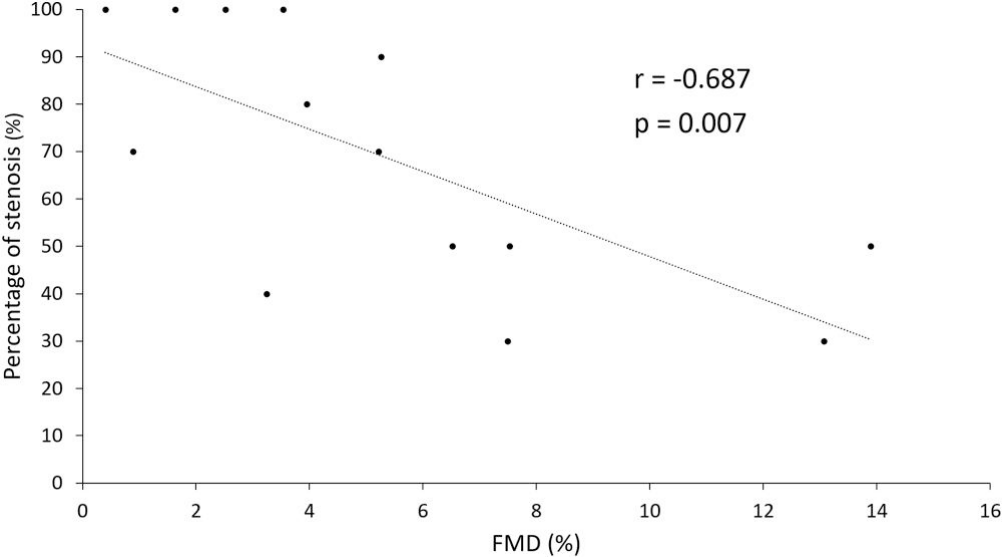
Correlation between FMD (%) and angiographically assessed coronary stenosis severity. FMD, flow-mediated dilation.

Each of the 14 patients referred to invasive coronary angiography had angiographic evidence of CAD. Five patients had 1-vessel, three patients 2-vessel, and six patients 3-vessel disease. The mean percentage of the most severe single stenosis observed was 69 ± 27%. Four patients had total occlusion in one of the main coronary arteries. There was a statistically significant negative correlation between FMD and the percentage of the most severe stenosis (*r* = −0.687, *P* = 0.007), as shown in *Figure 2*.

## Discussion

Our study demonstrated a statistically significant low to moderate correlation between FMD and adenosine-induced hyperaemic MBF as well as between FMD and the severity of CAD in invasive coronary angiography. To the best of our knowledge, the relation between peripheral endothelial function and quantitative MBF in patients with CAD has not been previously studied using (^15^O)H_2_O PET as the myocardial perfusion imaging modality.

Endothelium, a single layer of cells covering the surface of arteries, plays an important role in the maintenance of vascular health.^31^ One of the key functions of endothelium is the promotion of vascular dilation, mainly through the production of nitric oxide.^32^ Reactive hyperaemia following the release of arterial occlusion is the state-of-the-art method for assessing flow-mediated endothelial function. Endothelial dysfunction represents disturbance of the vasodilatory regulation and contributes to the development of atherosclerosis.^1^ Indeed, endothelial dysfunction is a precursor to atherosclerosis and is associated with traditional cardiovascular risk factors, such as hypercholesterolaemia, hypertension, diabetes mellitus, and smoking.^2–4,33,34^ Impaired endothelium-dependent arterial dilation can be observed even years before angiographic evidence of atherosclerosis appears.^35^

MBF is tightly regulated since myocardium is highly dependent on appropriate oxygen delivery. This regulation consists of several factors, including endothelial, metabolic, and neural.^36^ Nucleoside adenosine is considered critical in the metabolic aspect of this regulation.^37^ As a potent vasodilator, adenosine is thought to be a key contributor to coronary blood flow, especially in hypoxaemic and ischaemic conditions.^38,39^ Besides being an endogenous molecule, intravenously administered adenosine can be used to pharmacologically induce vasodilation during myocardial perfusion imaging.^40,41^ While several different adenosine receptor subtypes exist, the effect on coronary flow is considered to be mainly mediated through A_2A_ subtype, which is expressed in both endothelial and smooth muscle cells of coronary arteries.^42^ This indicates that adenosine-induced vasodilation is dependent on endothelium, but the subject has remained controversial. While some evidence shows that adenosine induces endothelium-dependent arterial vasodilation directly by a receptor-based mechanism,^19,20^ a couple of studies have not been able to confirm this.^21,22^ Cox *et al.*^23^ suggest that the effect of adenosine on endothelial cells might be indirect and caused by FMD. Thereby, although the vasodilation-regulating mechanism involved in using adenosine is complex, endothelium seems to be at least partially involved.

Myocardial perfusion PET imaging is the gold standard method for assessing MBF. While PET has excellent accuracy in determining the functional significance of epicardial atherosclerotic lesions,^43^ its utility goes even further by enabling the assessment of coronary microvascular function.^18^ Coronary microvascular dysfunction is a common term for several structural and functional disorders of the coronary microvasculature, one of which is endothelial dysfunction.^44^

Our study showed a low to moderate, statistically significant correlation between peripheral endothelial function and adenosine-induced hyperaemic MBF. Assessment of FMD is technically demanding, and the obtained result is associated with considerable variation due to biological and technical reasons. Thus, our result of a low or at most moderate correlation is not surprising. The statistical significance remained when controlling for sex, height, and diastolic blood pressure, which were possible confounding factors. This correlation is, in our opinion, quite logical, as peripheral endothelial dysfunction is connected to atherosclerotic disease. The results suggest that peripheral endothelial dysfunction is associated not only with atherosclerosis *per se* but also with the severity of CAD. This notion is reinforced by the inverse correlation between FMD and angiographically assessed CAD severity. However, based on the ROC analysis, FMD is not accurate enough to be used to diagnose reduced hyperaemic MBF. Thus, FMD measurement is not suitable for daily practice when assessing the possibility of ischaemia in patients with atherosclerotic disease.

The correlation between FMD and myocardial perfusion suggests that adenosine-induced MBF could function as a marker of coronary endothelial function. However, it is to be noted that all the patients had coronary atherosclerosis. Thus, it is likely that hyperaemic MBF was at least partly mediated by the flow-limiting effect of atherosclerosis, and it cannot be reliably differentiated from coronary endothelial function. Strengthening this assumption would require further studies including subjects with no atherosclerosis in epicardial coronary arteries.

Global resting MBF had no statistically significant correlation with FMD. This is not surprising, since patients with chronic and stable CAD and no history of myocardial infarction have normal MBF at rest. This was also true in our patients. No statistically significant correlation was observed between FMD and global MFR. This can be explained by the fact that MFR consists of the MBF determined at two different moments in time, and an index based on two separate measurements has more measurement uncertainty than one single measurement. Furthermore, MFR is a complex parameter because it does not exclusively describe maximum flow during pharmacological stress but is also influenced by MBF at rest.^45^

Previously, obesity has been associated with impaired coronary endothelial function.^46^ However, in the study by Sprung *et al.*,^47^ where FMD was used as the marker for endothelial function, metabolic syndrome, but not body mass, associated with endothelial dysfunction. In line, in our study population, neither weight nor body mass index showed significant correlations with FMD.

Torngren *et al*.^12^ reported a significant relationship between peripheral endothelial function (assessed with plethysmography) and coronary calcium score. In our study, we could not repeat their finding. This is somewhat surprising, since coronary calcification is considered as a specific marker of coronary atherosclerosis.^48^ On the other hand, FMD correlated significantly with the severity of angiographically assessed CAD. This suggests that impaired FMD is associated with the degree of artery disease, particularly in those with the most severe stenoses. This is in line with the study by Jambrik *et al*.^13^

The main limitation of this study is the rather small patient population. Due to limited capacity, we could not systematically examine all consecutive patients referred for CCTA *ad hoc* with FMD, but the subjects were invited for a separate visit. This limited the participation of those who live far away with poor transport connections. Although we tried to avoid excluding any patients except for those defined in the exclusion criteria, there may be some unintentional selection bias in our study population. Invasive coronary angiography was performed only in patients with significantly reduced MBF. Thus, comparison with angiographically determined CAD severity was only obtained for certain subpopulation. Regarding the degree of stenosis measured by CCTA, the limitation is the inaccuracy in determining the degree of severity related to the blooming artefact. Based on CCTA, all subjects had ≥50% stenosis at least in one epicardial coronary artery. Thus, based on CCTA, the study group was rather uniform and the CCTA findings did not allow a good division according to different severity of stenosis. An interesting set-up would be a comparison between healthy subjects and CAD patients to examine whether FMD is related to myocardial perfusion in patients without coronary atherosclerosis. However, PET imaging utilizes radioactivity, which limits the inclusion of healthy subjects. CCTA and PET imaging were performed consecutively on the same day, and the medications given during CCTA (metoprolol and isosorbide nitrate) might have influenced myocardial perfusion. Also, no restrictions on medication were set prior to FMD measurement, which could have affected the FMD results.

In conclusion, peripheral endothelial function is related with hyperaemic MBF and with the severity of CAD in invasive coronary angiography. Due to insufficient sensitivity and specificity in the identification of reduced MBF, FMD is not suitable for clinical practice at the individual level. However, it works at the population level as a research tool when assessing endothelial dysfunction in patients with CAD.

## Lead author biography


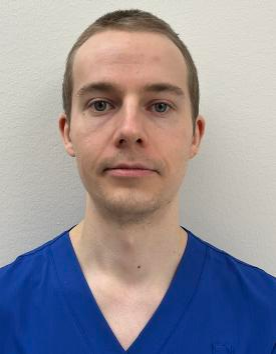
Niklas Vartiainen is a physician and a specialist in clinical physiology and nuclear medicine. He works at the Kuopio University Hospital and is also conducting research for his doctoral thesis.

## Consent

The study protocol was approved by the Ethics Committee of the Northern Savo Hospital District. Prior to participation, the patients were informed of the study protocol, and they provided their informed consent.

## Data Availability

The data underlying this article cannot be shared publicly because the permission to use this data is restricted by the General Data Protection Regulation (EU) 2016/679. The outputs of statistical analyses are available by request to the corresponding author.
